# Effect of Chemically Engineered Au/Ag Nanorods on the Optical and Mechanical Properties of Keratin Based Films

**DOI:** 10.3389/fchem.2020.00158

**Published:** 2020-03-10

**Authors:** Marta Gambucci, Annalisa Aluigi, Mirko Seri, Giovanna Sotgiu, Giulia Zampini, Anna Donnadio, Armida Torreggiani, Roberto Zamboni, Loredana Latterini, Tamara Posati

**Affiliations:** ^1^Dipartimento di Chimica, Biologia e Biotecnologie, Università di Perugia, Perugia, Italy; ^2^Consiglio Nazionale delle Ricerche, Istituto per la Sintesi Organica e la Fotoreattività (CNR-ISOF), Bologna, Italy; ^3^Dipartimento di Scienze Farmaceutiche, Università di Perugia, Perugia, Italy

**Keywords:** plasmonic nanorods, keratin, films, biocomposites, optical properties, mechanical properties

## Abstract

In this work we report the preparation and characterization of free-standing keratin-based films containing Au/Ag nanorods. The effect of nanorods surface chemistry on the optical and mechanical properties of keratin composite films is fully investigated. Colloid nanorods confer to the keratin films interesting color effects due to plasmonic absorptions of the metal nanostructures. The presence of metal NRs induces also substantial change in the protein fluorescence emission. In particular, the relative contribution of the ordered-protein aggregates emission is enhanced by the presence of cysteine and thus strictly related to the surface chemistry of nanorods. The presence of more packed supramolecular structures in the films containing metal nanorods (in particular cysteine modified ones) is confirmed by ATR measurements. In addition, the films containing nanorods show a higher Young's modulus compared to keratin alone and again the effect is more pronounced for cysteine modified nanorods. Collectively, the reported results indicate the optical and mechanical properties of keratin composites films are related to a common property and can be tuned simultaneously, paving the way to the optimization and improvement of their performances and enhancing the exploitation of keratin composites in highly technological optoelectronic applications.

## Introduction

The preparation of innovative and multifunctional bionanocomposite free-standing films starting from natural polymers and nanosized metals represents an attractive challenge for several applications including packaging, catalysis, optics, biomedicine, tissue engineering, drug delivery, and electronics (Ruiz-Hitzky et al., 2010). The added value of nanocomposites is represented by the synergistic properties arising from the combination of different materials with peculiar features, such in the case of bioderived and inorganic components.

Among biopolymers, keratin is a promising candidate for engineering innovative bionanocomposites for biomedical as well as technological applications. Indeed, keratin is one of the most abundant renewable and non-food proteins, being the principal component of hair, wool, feather, horns and nails of mammals, reptiles, and birds. This protein can be easily extracted from readily available and low-cost wastes, such as raw wool not suitable for spinning, feathers from butchery, and by-products of the wool textile industry (Aluigi et al., 2007). Regenerated keratin solutions extracted from wool can be easily processed in different biodegradable, biocompatible and non-toxic forms including fibers, capsules, particles, gels, foams, nanofibrous electrospun membranes, and films (Wang et al., 2017; Posati et al., 2018; Giuri et al., 2019). So far keratin based materials have attracted a lot of attention in biomedical applications such as porous scaffolds for tissue engineering, wound healing, and substrates for *in vitro* studies (Rouse and Van Dyke, 2010). Only recently, keratin films have been also investigated as innovative natural support material for a new generation of sustainable and greener electronic and optoelectronic devices (Natali et al., 2019; Posati et al., 2019).

Nanosized metals are extensively studied because of their excellent spectroscopic, electronic, and chemical properties arising from the combination of the small size with a large surface energy (Kelly et al., 2003; Harish et al., 2018). In particular, plasmonic metals nanoparticles such as gold (Au) and silver (Ag) can provide interesting biomedical and/or optical properties and the size, morphology, stability, and surface chemistry of these nanomaterials can strongly influence their performances. Plasmonic NPs are widely studied for their strong scattering, absorption of light and fluorescence emission, enabling their application in several fields such as labeling, imaging, and electronics. In particular, the efficiency enhancement of optoelectronic devices, such as organic light emitting diodes (OLEDs) (Munkhbat et al., 2016; Feng et al., 2018), organic solar cells (Li et al., 2012; Vangelidis et al., 2018), and light-detectors (Xie et al., 2017), have been largely demonstrated and ascribed to the effect of their peculiar optical properties.

Beside the aforementioned properties, Au and AgNPs have a strong binding affinity for ligands containing –SH, –NH_2_, and –COOH functional groups (Luo, 2007), which are also present in proteins such as fibroin, keratin etc. Indeed, several works report the preparation of stable Ag and Au NPs using extracted wool keratin as capping agent (Lü and Cui, 2010; Shanmugasundaram and Ramkumar, 2018), in order to combine their antibacterial properties for wound-healing applications. Diversely, only few reports are focused on the preparation of keratin–plasmonic NPs composites films (Tran et al., 2016, 2018) and on their potential application in electronic devices. All these works confirm how the combination of polymeric matrices such as proteins with inorganic nanoparticles can allow the addition of specific and tunable functionalities (optical, magnetic and electrical properties, etc). It is worth also mentioning that for technological applications, beside the possible active roles of dispersed NPs in a matrix, their presence can simultaneously contribute to the enhancement of the mechanical properties of the resulting nanocomposites films (Posati et al., 2014). Nevertheless, nowadays a detailed study on the correlation between the mechanical and optical properties of a given hybrid nanocomposite system is still missing.

In light of this, we report here the preparation and characterization of keratin-based films containing Au and Au/Ag nanorods using citrate or cysteine as capping agents. The dispersion of Au and Au/Ag nanostructures allows to significantly alter the optical properties of the material without modifying considerably the morphology of the nanomaterials. On the other hand, since cysteine is an amino acid and has the tendency to create networks on its own, its addition on the nanorods surface is likely to promote the interaction with (and the binding to) the keratin structure. The influence of the nanomaterials surface chemistry on the mechanical and optical properties of keratin composite films was correlated and fully investigated by different techniques. The metal colloids have been prepared through controlled reduction processes in order to tightly regulate some of their characteristics, such as shape (Latterini and Tarpani, 2016) and surface chemistry (Gambucci et al., 2018). As a result, the successful preparation of different keratin-based nanocomposites films and their detailed characterization allowed to identify the key aspects to further optimize/modulate the resulting optical and mechanical properties, thus enhancing the exploitation of keratin nanocomposites as smart and multifunctional materials/components (e.g., supports) for sustainable and greener optoelectronic devices.

## Experimental Section

### Materials

High molecular weight keratin powder (~50 kDa) is kindly donated by Kerline srl. All chemicals are of analytical grade and purchased from Sigma-Aldrich. Gold (III) chloride trihydrate (HAuCl_4_·3H_2_O ≥ 99.9 %, Sigma Aldrich), silver nitrate (AgNO_3_ 99%, Sigma Aldrich), hexadecyltrimethylammonium bromide (CTAB ≥ 98%, Sigma Aldrich), sodium borohydride (NaBH_4_ 98%, Sigma Aldrich), L-ascorbic acid (reagent grade, Sigma Aldrich), Poly(sodium 4-styrenesulfonate) (Na-PSS average Mw ≈70.000, Sigma Aldrich), tri-sodium citrate (99%, Fluka), L-cysteine (≥ 97%, Sigma Aldrich) are all used without further purification. Nanopure water (≤ 15.0 MΩ) from a Millipore Milli-Q gradient system is used as solvent.

### Methods

#### Synthesis of Nanorods (NRs)

##### Synthesis of gold nanorods (AuNRs)

Citrate-stabilized gold nanorods (AuNRs) have been prepared through the seed-mediated method following the literature procedure (Nikoobakht and El-Sayed, 2003), with minor modifications. Gold seeds are prepared by adding 0.6 mL of an ice-cooled NaBH_4_ solution (10 mM) to a 0.25 mM HAuCl_4_ solution in 0.1 M CTAB. Then, 48 μL of the as-prepared seed-solution are quickly injected into 41 mL of a 27°C-thermostated growth solution (0.5 mM HAuCl_4_, 0.08 mM AgNO_3_, 0.078 M L-ascorbic acid and 0.1 M CTAB) and left under stirring at 27°C for 2 h. The obtained CTAB-AuNRs are then washed with Milli-Q water to remove the excess of CTAB and the eventual residues of the synthetic procedure.

To change the stabilizer from CTAB to citrate, multiple centrifugation/redispersion cycles are carried out (Mehtala et al., 2014). Briefly, 10 mL of the CTAB stabilized AuNRs suspension are centrifuged and the pellet obtained is re-dispersed in 0.15 wt% Na-PSS; the procedure is repeated twice to ensure the complete removal of CTAB and its substitution with Na-PSS. After the second centrifuge, the pellet is then dispersed in 10 mL of 5 mM tri-sodium citrate yielding the final AuNRs sample.

The gold concentration in the final AuNRs sample, evaluated through ICP analysis (see below), results to be 3.025 × 10^−4^ M, which gives an estimated concentration of nanorods of 6.3 × 10^−10^ M.

##### Synthesis of silver-coated gold nanorods (Au@AgNRs)

Six milliliter of the citrate-capped AuNRs suspension are placed, under vigorous stirring, in 2.7 mL of 1 mM AgNO3 solution; then 0.9 mL of a 0.1 M L-ascorbic acid solution are added dropwise and the mixture is left under stirring for 30 min, resulting in the sample Au@AgNRs.

Through ICP analysis it was possible to assess the concentration of both gold and silver, equal to 1.168 × 10^−4^ M and 7.090 × 10^−5^ M respectively, from which it has been derived a concentration of nanorods equal to 2.10 × 10^−10^ M (see Supplementary Material for further details).

##### Preparation of cysteine-stabilized nanorods (AuNRs_*cyst*_ and Au@AgNRs_*cyst*_)

In order to obtain cysteine-stabilized nanorods, to each aqueous NRs suspension (AuNRs or Au@AgNRs), a weighted amount of solid cysteine was added to obtain a final cysteine concentration of 50 mM. The system was stirred until the complete solubilization of cysteine and afterwards the sample was stored at 4°C. All aqueous NRs suspension showed a pH value of about 6.5.

#### Preparation of Keratin and Keratin/NRs Hybrid Films

Keratin was extracted by Merino wool (21 μm), through the sulfitolysis reaction, as described in the supporting information and in reference (Posati et al., 2019). The keratin films (KF) were prepared by casting the keratin aqueous solution (10% wt) containing glycerol as plasticizer (25% wt vs. keratin). The amount of the different NRs (AuNRs, Au@AgNRs, AuNRs_cyst_, and Au@AgNRs_cyst_) was 0.2% wt vs. keratin.

#### Characterization of Keratin and Keratin/NRs Hybrid Films

As previously described in reference (Posati et al., 2019), infrared spectra were acquired using the attenuated total reflectance technique (ATR) with a Bruker Vertex 70 interferometer equipped with a diamond crystal single reflection Platinum ATR accessory, in the 4,000–600 cm^−1^ region, with 100 scans and a resolution of 4 cm^−1^. The FTIR analysis were carried out on spectra normalized for the reference band at 1,650 cm^−1^ (Amide I adsorption). In order to study the protein secondary structure, the spectra in the 1,480–1,780 cm^−1^ region were corrected by means of a weighted subtraction of the water spectrum, and smoothed with an eleven point Savitsky-Golay function. Resolution of the amide I and II bands was done by means of Gaussian shape related to different protein secondary structures. The fitting procedure was done with the ORIGIN 8.1 (OriginLab Corporation, MA, USA) defining the peak numbers by second order derivative analysis of the spectra. The results of the fitting procedure were further evaluated by investigating the residual from the difference between fitted and original curve and accepted when the *R*^2^ value was higher than 0.99. The mechanical properties of the films were determined by the tensile tests following the same procedure described in reference (Posati et al., 2019), with a Zwick Roell Z1.0 testing machine, with a 200 N static load cell using at least three replicates. For that, the films were cut in rectangles of 5 mm width and length 6 cm and fixed on the grips of the device with a gap of 20 mm. The thickness of the sample, determined with an uncertainty of ± 5 μm, was in the range 90–100 μm. The films were tested at a speed of 30 mm min^−1^. The data were elaborated by the TestXpert V11.0 Master software (Boaretti et al., 2018). The Young's modulus (E) was calculated from the linear part of the stress–strain curve, between 0.2 and 0.6% elongation. The standard deviation, resulting from the analysis of at least three film stripes for each sample, was considered for statistical analysis.

The optical properties of the colloidal samples and the films are investigated through a Cary 8454 UV-VIS Diode Array spectrophotometer, whereas the corrected fluorescence emission and excitation spectra are acquired through an Edinburgh Instrument FS5 Spectrofluorometer, equipped with a sample-holder for solid and liquid samples.

Varian 700-ES series ICP-OES spectrometer is used to determine the metal concentrations. For each sample, a weighted amount on nanorods is dissolved in aqua regia (HCl/HNO_3_ 3:1) and then diluted with MilliQ water before performing the measurement.

The morphology of both AuNRs and Au@AgNRs is investigated by a Philips 208 transmission electron microscope (TEM) following the same procedure described in reference (Zampini et al., 2017) operating at 80 kV of beam acceleration. For each sample, a drop of the aqueous colloidal solution was deposited on a 300 square mesh Formvar-coated copper grid.

The morphology of the films and the elemental analysis of metals was conducted with a scanning electron microscope (SEM, ZEISS EVO LS10) fitted with an EDS detector (Bruker mod. Quantax System). For the analysis of films sections, the samples were frozen in liquid nitrogen and then broken in order to induce a fragile fracture.

## Results and Discussion

### Morphological and Optical Properties of Nanorods Suspensions

**Figure 1:**
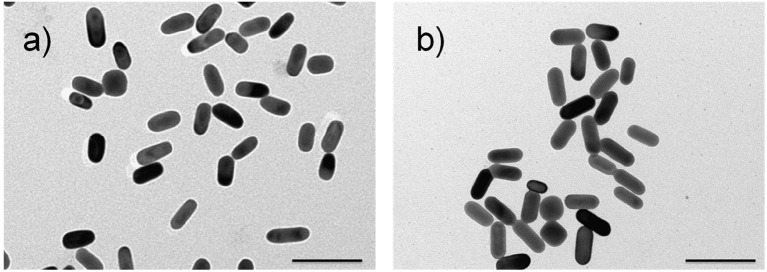
Representative TEM images of AuNRs **(a)** and Au@AgNRs **(b)** samples. Scale bars: 100 nm.

**Figure 2:**
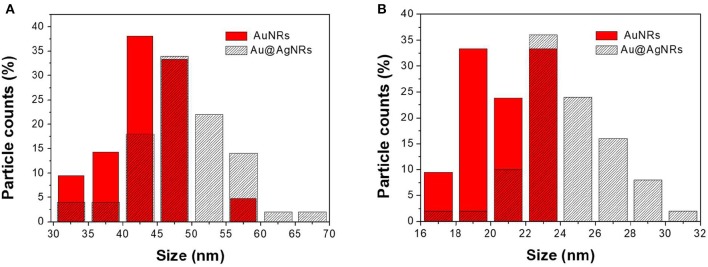
Size distributions of AuNRs and Au@AgNRs length **(A)** and width **(B)**.

The adopted synthetic procedures for nanorods have been selected with the aim to substantially modify the optical properties of the nanomaterials without significantly modifying their morphology. The seed-mediated synthesis of gold nanostructures assisted by CTAB and Ag^+^/Ag^0^, should result in an asymmetric and controlled growth. TEM imaging (Figure 1a) documents that the procedure generates gold nanorods (AuNRs) sample with an aspect ratio of about 2.0, in agreement with literature reports (Nikoobakht and El-Sayed, 2003; Lohse and Murphy, 2013). TEM images recorded on Au@AgNRs sample, shows rod-like structures with a homogeneous contrast, suggesting that silver is deposited on the gold nanostructures (no isolated silver nuclei can be observed) forming an uniform layer without significantly alter the dimension and the morphology of the rod (Figures 1b, 2). Actually the dimensional analysis of the TEM images (Figure 2), before and after silver deposition, enables to estimate the thickness of the silver layer is about 1–2 nm. However, the optical properties of the metal nanomaterials are considerably different. Generally, nanorods samples present extinction spectra characterized by two main bands, generated by the transversal, and longitudinal surface plasmonic resonance (SPR), due to the resonant oscillations of the conductive electrons of the material induced by the incident radiation. The frequencies of these oscillations are strongly dependent on the chemical nature of the colloids and their environment.

**Figure 3:**
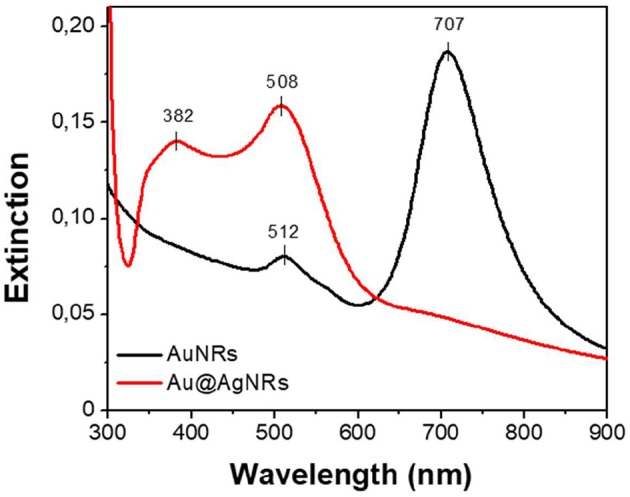
Extinction spectra of AuNRs (black) and Au@AgNRs (red) aqueous suspensions.

As reported in Figure 3, the bands associated to AuNRs (black line) are centered at 512 and 707 nm for the transversal and longitudinal components, respectively. When AuNRs are homogeneously covered by a silver layer (Au@AgNRs) the SPR bands shift to 382 nm and 508 nm (red line) characteristic of the silver SPR (Lamri et al., 2018). In such way, different optical responses are obtained, preserving the nanostructure dimension distribution. In order to modify the affinity between the nanorods and the keratin protein, a fixed amount of cysteine (50 mM) is added at the end of the synthetic procedure, generating two additional samples named AuNRs_cyst_ and Au@AgNRs_cyst_.

### Keratin Film Preparation and Optical Characterization

**Figure 4:**
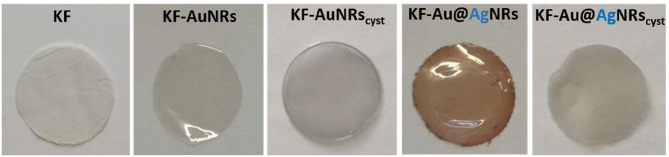
Picture of keratin films containing different nanorods.

**Figure 5:**
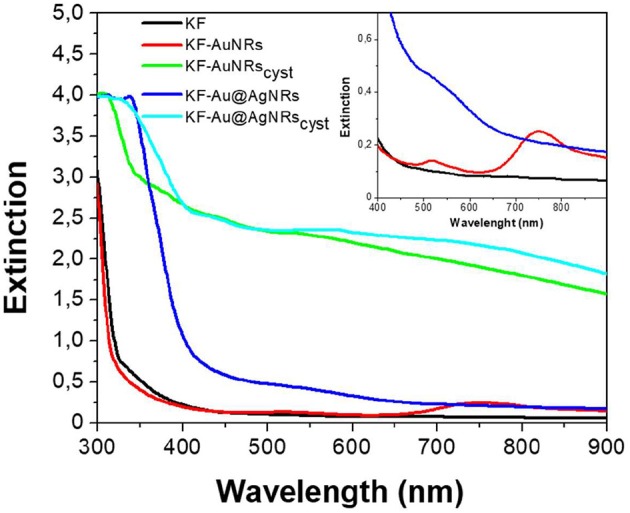
Extinction spectra of keratin-based solid films in absence (black) and in presence of AuNRs (red), Au@AgNRs (blue), AuNRs_cyst_ (green), and Au@AgNRs_cyst_ (cyan) in concentration of 0.2% wt vs. keratin. Inset: magnification.

Keratin free-standing films (KFs) with a thickness of ~90 μm (measured by micrometer) were prepared according to the same procedure described in reference (Posati et al., 2019). Specifically, all films were obtained by casting keratin aqueous solution containing glycerol as plasticizer, on polystyrene substrate in order to reduce the adhesion between keratin and support, thus favoring the removal of the resulting film. Different types of nanorods (AuNRs, Au@AgNRs, AuNRs_cyst_, and Au@AgNRs_cyst_) were added to the keratin solutions, in order to obtain NRs-modified KFs (Figure 4). Non-modified KF is slightly yellowish and presents good transparency in the visible range (Figures 4, 5, black line). On the other hand, when nanorods colloids are dispersed in the protein matrix, the films show interesting color effects (Figure 4). Indeed, AuNRs confer a brownish color to the film (KF-AuNRs), with plasmonic bands centered at 518 and 749 nm (Figure 5, red line), whereas Au@AgNRs (blue line) present blue-shifted bands which result in a dark yellowish color. It has to be noted that despite the color due to plasmonic absorptions of the metal nanostructures, although less intense than the suspensions due to the dilution effect, the films retain good optical transparency. The situation dramatically changes when cysteine is present. Indeed, both KF-AuNRs_cyst_ (Figure 5, green line) and KF-Au@AgNRs_cyst_ (Figure 5, cyan line) samples show different color shades and an evident reduction of the transparency compared to KF-AuNRs and KF-Au@AgNRs. This effect might be due to cysteine, which can alter the protein network with a possible chains aggregation.

The dispersion of NRs into the protein matrix was investigated by scanning electron microscopy (SEM) analysis. The SEM images (Figure S1) of the internal microstructure, obtained with a cryo-fractured samples, shows that both types of AuNRs and Au@AgNRs nanostructures are quite homogeneously distributed throughout the keratin film with the presence of few aggregates having dimension of few hundreds of nanometers. In addition, EDS analysis confirms that these structures consist of Au or Au@Ag.

**Figure 6:**
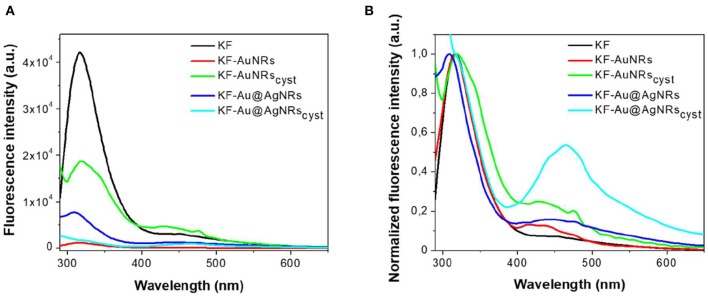
Fluorescence emission spectra **(A)** and normalized fluorescence emission spectra **(B)** acquired exciting at 280 nm keratin-based solid films in absence (black) and in presence of AuNRs (red), Au@AgNRs (blue), AuNRs_cyst_ (green), and Au@AgNRs_cyst_ (cyan) in concentration of 0.2% wt vs. keratin.

**Figure 7:**
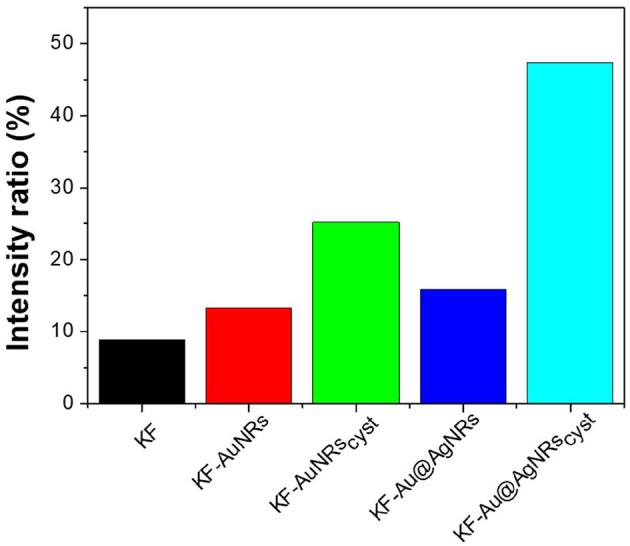
Intensity ratio% of the main emission bands generated by different keratin-based films.

The impact of Au/Ag NRs and cysteine on the protein films has been further investigated through fluorescence measurements (Figure 6). For all samples, included KF, the fluorescence spectra are characterized by two main emission bands: the first one, between 300 and 400 nm, is attributed to the natural emission of aromatic amino acids such as tryptophan and tyrosine in monomeric form (Lakowicz, 2006); the second one at lower-energy (400–500 nm), can be assigned to keratin's ordered-fiber aggregates, as a result of π-π interactions among amino acids in the film (Chan et al., 2013). As shown in Figure 6A, the presence of nanorods in keratin films does not significantly modify the position of the fluorescence bands, but induces a substantial change in their relative intensities. Independently from the fraction of absorbed light, fluorescence intensity of the monomer decreases compared to the visible band assigned to the protein aggregates (Figure 6B). The effect on the relative emission intensities, which can be quantified by intensity ratio (I_450_/I_315_ × 100), depends on the chemical nature of the nanorods and their surface functionalization; in particular, relative contribution of the aggregate's emission is enhanced in the presence of cysteine (Figure 7). Looking at the results shown in Figure 7, it is evident that the presence of nanorods leads to an increase of the macromolecular packing of the system. Indeed, the intensity ratio determined for the rod-keratin films is always higher than that of pure KF, thus the contribution of the Vis band is higher in the presence of metal colloids. Moreover, the presence of cysteine on the surface of metal colloids, increases the order of the system, doubling or even tripling the contribution of the Vis band due to the ordered-structure emission. Finally, silver especially when associated with cysteine, seems to strongly influence the protein chains packing, giving a contribution even 5-times higher than that of the pure keratin film.

### ATR Characterization

**Figure 8:**
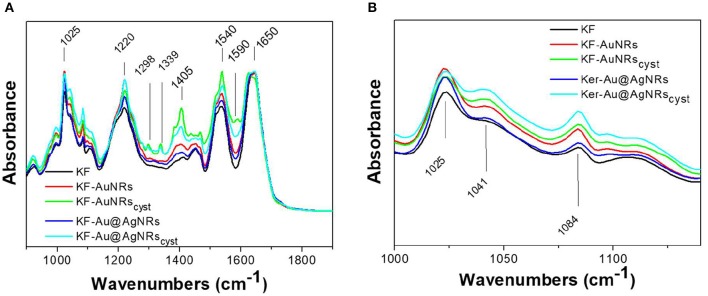
ATR spectra of the prepared samples **(A)** in the 2,000–900 cm^−1^ region and **(B)** in the 1,000–1,200 cm^−1^ region.

**Figure 9:**
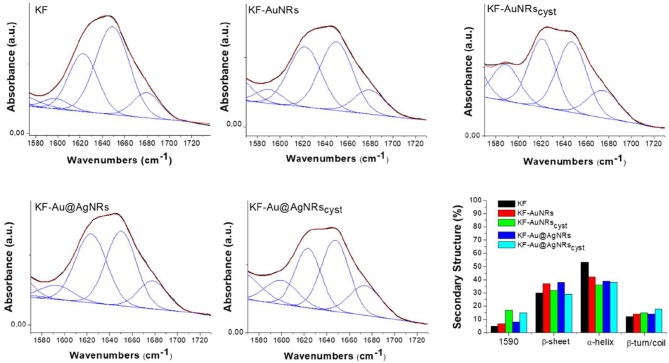
Amide I curve fitting of keratin films and percentages of keratin secondary structures. Black line: Experimental Curve; blu lines: Fitting peaks and Red line: Resulting fitting curve.

The ATR spectra of the keratin films loaded with the different nanorods and normalized for the reference band at 1,650 cm^−1^ are shown in Figure 8. All the spectra show the characteristic adsorption bands of the peptide bonds (-CONH-). Particularly, in the spectral region between 2,000 and 890 cm^−1^, the peptide vibrations originate bands known as Amides I, II and III. The Amide I connected mainly to the C=O stretching, has its peak at 1,650 cm^−1^; the Amide II band, which falls at 1,540 cm^−1^ is mainly related to the N-H vibration; while the Amide III band (1,230 cm^−1^) is referred to an in phase combination of C-N stretching and N-H in plane bending, with some contribution of the C-C stretching and C-O bending vibration (Wojciechowska et al., 1999). In order to study the protein structural changes induced by the metal nanorods, the amide I of each sample was resolved into Gaussian bands (Figure 9) whose number was defined in the second order derivative spectrum (data not shown). The secondary structure assignments (based on the band positions) and the relative percentage (based on the band area percentage) were reported in Figure 9. All the films had contribution of different conformations as β-turn/disordered (1,685–1,671 cm^−1^), α-helix (1,650–1,657 cm^−1^), and β-sheet (1,631–1,621 cm^−1^) structures (Cardamone, 2010). As well-known, for the keratin film regenerated from water, the percentage of α-helix structures (53%) is higher than that of β-sheet structures (30%) (Aluigi et al., 2007). As can be seen, the addition of metal nanorods induces a significant decrease of α-helix structures, associated to a slight increase of more packed β-sheet and β-turn/coil structures.

Furthermore, the ATR spectra of the film made with cysteine capped nanorods show additional bands related to the cysteine (Figure 8A) such the band at 1,590 cm^−1^ (C=O), 1,405 cm^−1^ (CH_2_ and CH_3_ bending), 1,298 and 1,339 cm^−1^ (C-O) (Devi et al., 2016). These bands are more intense for the KF-AuNRs_cys_ than for the KF-Au@AgNRs_cys_, probably for a greater amount of nanorods on the surface of keratin based film.

Finally, the strong band at 1,025 cm^−1^ is due to the S-O stretching vibration of the Bunte salt ($-SSO_{3}^{-}$. In Figure 8B, the 1,000–1,200 cm^−1^ region of S-O stretching vibration is shown. As can be seen, compared to pristine keratin, the sample with the nanorods showed enhanced absorption bands at 1,041 cm^−1^ and 1,084 cm^−1^ associated to the sulphonates ($SO_{3}^{-}$ in cysteic acid and to the cystein mono-oxide (-S-SO-), respectively, likely due to higher affinities of these functional groups for the metal surfaces (Mun et al., 2019; Semenyshyn et al., 2019). The presence of these groups might increase the weak intermolecular interaction between the protein chains leading to a partial aggregation of the system, at least locally.

### Mechanical Properties of KF-NRs

**Figure 10:**
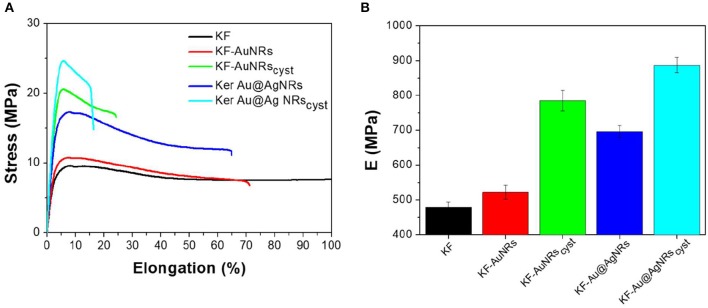
**(A)** Stress–strain data and **(B)** E modulus of the different KF samples.

Stress–strain mechanical tests were performed, at room temperature, on samples conditioned at 33% RH for a week. Table 1 shows that the mechanical properties of KF films are affected in the whole by the presence of nanorods with different surface functionalization. The stress-strain curve profile (Figure 10A) shows an increase in the tensile stress as well as to a significant reduction in the elongation at break for composites containing cysteine. This is probably due to a lower molecular mobility of the ordered β-sheet structure, induced by the presence of nanostructures. Cysteine is able to well-interact with keratin and to be incorporate into the protein matrix leading to a chain stiffness. Thus, a higher toughness (W, more than 50%) was obtained for composites based on keratin-and cysteine capped NRs. The mechanical data suggest that the presence of Ag layer on the Au nanorods contributes to increase the mechanical parameters of keratin composites in comparison with pristine AuNRs.

**Table 1:** Mechanical parameters of the different KF samples.

**Sample**	**E (MPa)**	**Stress max (MPa)**	**W at Stress max (J/cm^**2**^)**	**Strain **(*%*)****
KF	479 ± 16	10.8 ± 0.9	0.14 ± 0.01	90 ± 7
KF-AuNRs	522 ± 20	10.7 ± 0.8	0.14 ± 0.01	71 ± 7
KF-AuNRs_cyst_	785 ± 30	20.0 ± 0.7	0.17 ± 0.01	24 ± 4
KF-Au@Ag NRs	696 ± 17	17.2 ± 0.7	0.21 ± 0.01	64 ± 7
KF-Au@Ag NRs_cyst_	887 ± 21	24.6 ± 0.7	0.22 ± 0.01	15 ± 4

Fluorescence emission spectra showed that in the keratin composite films the presence of nanorods, especially those stabilized with cysteine, leads to an increase of ordered-protein aggregates (Chan et al., 2013). As indicated by ATR measurements these ordered aggregates could be the result of (i) an increase of the intermolecular interaction between the protein chains due to the high affinities of (${\text{SO}}_{3}^{-}$) and (-S-SO-) functional groups for the metal surfaces and/or (ii) a decrease of α-helix structures associated to a slight increase of β-sheet and β-turn/coil structures. As reported for other similar proteins (Sagnella et al., 2016), the β-sheet or in general more packed supramolecular structures have a higher stiffness than α-helix and random coil structures, therefore the composite films based on cysteine show actually a higher modulus with respect to those containing only metal nanorods (Figure 10B).

## Conclusion

Free-standing keratin films containing Au/Ag NRs were prepared by drop-casting, starting from an aqueous protein solution doped with nanorods and containing glycerol as plasticizer. The effect of nanorods surface chemistry on the optical and mechanical properties of keratin composite films was investigated, by modifying AuNRs surface with Ag coating and/or addition of cysteine. Keratin composites films showed interesting color effect due to the plasmonic absorption of the metal colloids and a good transparence in the UV Vis region except for the samples containing cysteine modified NRs. Fluorescence measurements indicated that the presence of NRs in keratin films did not significantly modify the position of the fluorescence bands, but induced a substantial change in their relative intensities. In particular, the relative contribution of the ordered proteins aggregate emission was enhanced in the presence of NRs, especially cysteine-modified ones, suggesting that the presence of NRs led to an increase of the macromolecular packing of the system. This protein chains aggregation was confirmed by ATR measurements. In fact, in the keratin composites were observed both an increase of the intermolecular interaction between the protein chains due to the high affinity of sulphonates and cystein mono-oxide groups for the metal surfaces and a decrease of α-helix structures associated to a slight increase of β-sheet and β-turn/coil structures. Moreover, in agreement with the fluorescence emission data, the composites films showed a higher Young's modulus compared to keratin alone, in particular when cysteine is present. In conclusion, we observed a similar trend in the optical and mechanical properties of keratin composites films and we identified the key aspects to prepare tailored innovative keratin-plasmonic NRs materials with specific features suitable for technological optoelectronic applications.

## Data Availability Statement

All datasets generated for this study are included in the article/Supplementary Material.

## Author Contributions

MG made the synthesis of nanorods and collected the laboratory experimental data (optical measurements). AA contributed to the ATR characterization and data discussion. MS contributed to the data discussion and paper revision. GS made keratin extraction and purification. GZ was involved in the synthesis of nanorods and in the ANALYSIS of laboratory experimental data. AD carried out mechanical tests and discussed mechanical data. AT is the leader of the RM @ School project, which funded the publication of this work, gave a great support for the ATR characterizations, and contributed to the paper revision. RZ contributed to the data discussion and to the paper revision. LL worked on the study design and writing and coordinated the data collection and discussion for the realization of this work. TP prepared keratin composites films, worked on the study design and writing, and coordinated the data collection and discussion for the realization of this work.

### Conflict of Interest

The authors declare that the research was conducted in the absence of any commercial or financial relationships that could be construed as a potential conflict of interest.
