# Supplementary material for: Effect of Chemically Engineered Au/Ag Nanorods on the Optical and Mechanical Properties of Keratin Based Films
Source: Front Chem. 2020 Mar 10;8:158. doi: 10.3389/fchem.2020.00158 (PMC7078657; doi:10.3389/fchem.2020.00158)
Supplement: Supplementary file 1 [file Data_Sheet_1.docx]

# Supplementary Material

**Effect of chemically engineered Au/Ag nanorods on the optical and mechanical properties of keratin based films**

**Marta Gambucci,^1^ Annalisa Aluigi,^2^ Mirko Seri,^2^ Giovanna Sotgiu,^2^ Giulia Zampini,^1^ Anna Donnadio,^3^ Armida Torreggiani,^2^ Roberto Zamboni,^2^ Loredana Latterini*^1^ and Tamara Posati*^2^**

^1^ Dipartimento di Chimica, Biologia e Biotecnologie, Università di Perugia, Via Elce di Sotto, 8- 06123 Perugia, Italy.

^2^ Consiglio Nazionale delle Ricerche, Istituto per la Sintesi Organica e la Fotoreattività (CNR-ISOF), via Piero Gobetti 101, 40129 Bologna, Italy.

^3^ Dipartimento di Scienze Farmaceutiche, Università di Perugia, Via del Liceo, 1, 06123 Perugia, Italy.

*** Correspondence:**Corresponding Author
Tamara Posati: [tamara.posati@isof.cnr.it](mailto:tamara.posati@isof.cnr.it)

Loredana Latterini: loredana.latterini@unipg.it

**- Section I. Keratin extraction from wool fibres**

A fibre sample, withdrawn from a combed sliver and cleaned by Soxhlet extraction with petroleum ether, was washed with distilled water and dried at 21°C and 60% relative humidity overnight. Keratin was extracted from wool by sulfitolysis reaction. Briefly, cleaned fibres (5g) were cut into snippets and dispersed in 100 ml of aqueous solution containing urea (8M), sodium metabisulphite (0.5M) and sodium dodecyl sulphate (SDS, 0.1M), under mechanical shaking at 65°C overnight. The mixture was filtered with vacuum filter (10-16 µm cut off), dialyzed against distilled water using a cellulose tube (molecular weight cut-off 12-14 kDa) for 3 days at room temperature, changing the distilled water four times a day. The resulting aqueous solution was freeze-dried in order to obtain pristine keratin powder.

During the sulfitolysis, cysteine disulphide bonds are cleaved by sulphite ions to give reduced keratin (WS^-^) and cysteine-S-sulphonate keratin (${WSSO}_{3}^{-}$), according to the reaction:

$$WSSW+ {SO}_{3}^{2-}\to{WS}^{-}+ {WSSO}_{3}^{-}$$

WSSW is the cross-linked wool keratin, WS^-^ is the reduced keratin and $\mathrm{WSSO}_{3}^{-}$ is the cystein-S-sulphonated keratin.

**- Section II. ICP analysis and calculations**

AuNRs concentration was determined through eq.1:

| $\left[ AuNRs \right]=\frac{\left[ Au \right]_{0}}{N_{atoms}}$ | (1) |
| --- | --- |

where [Au]_0_= [Au]_ICP_*(Dilution factor), while N_atoms_ is calculated as:

| $N_{atoms}= \frac{V_{particle}}{V_{Au atom}}$ | (2) |
| --- | --- |

In eq. 2, a cylindrical geometry was considered for AuNRs. The concentration of Au@AgNRs has been calculated in a similar way.

**Section III. Additional figures**


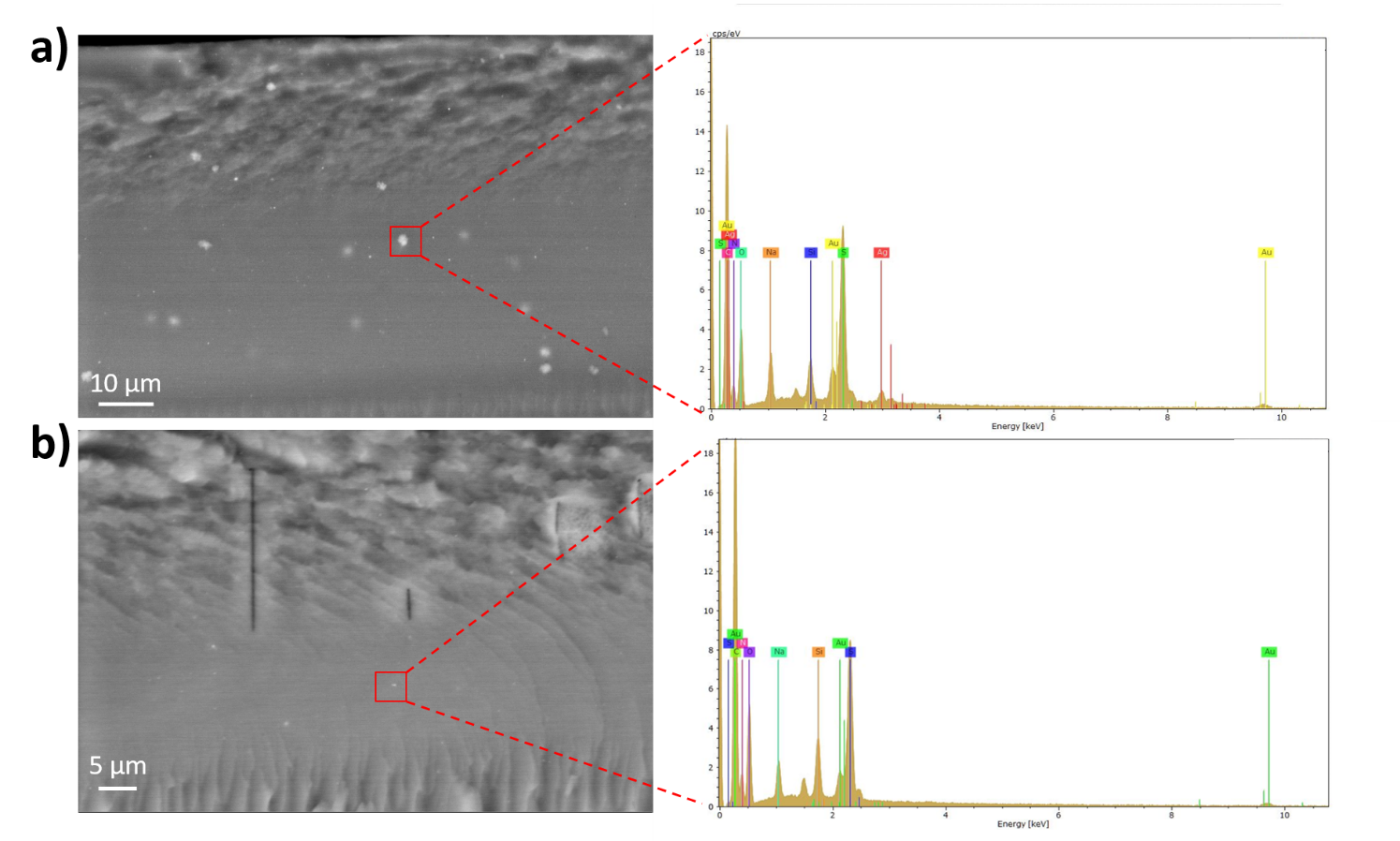


**Figure S1:** SEM images (left) and EDS analysis (right) of a) KF-Au@AgNRs and b) KF-AuNRs samples.
